# Do social identity and cognitive diversity correlate in environmental stakeholders? A novel approach to measuring cognitive distance within and between groups

**DOI:** 10.1371/journal.pone.0244907

**Published:** 2021-11-04

**Authors:** Payam Aminpour, Heike Schwermer, Steven Gray

**Affiliations:** 1 Department of Community Sustainability, Michigan State University, East Lansing, MI, United States of America; 2 Collective Intelligence Research Group, IT University of Copenhagen, København, Denmark; 3 Institute of Marine Ecosystem and Fishery Science, Center for Earth System Research and Sustainability, University of Hamburg, Hamburg, Germany; 4 Department of Economics, Center for Ocean and Society, Christian-Albrechts-University Kiel, Kiel, Germany; Stockholms Universitet, SWEDEN

## Abstract

Groups with higher cognitive diversity, i.e. variations in how people think and solve problems, are thought to contribute to improved performance in complex problem-solving. However, embracing or even engineering adequate cognitive diversity is not straightforward and may even jeopardize social inclusion. In response, those that want to promote cognitive diversity might make a simplified assumption that there exists a link between identity diversity, i.e. range of social characteristics, and variations in how people perceive and solve problems. If this assumption holds true, incorporating diverse identities may concurrently achieve cognitive diversity to the extent essential for complex problem-solving, while social inclusion is explicitly acknowledged. However, currently there is a lack of empirical evidence to support this hypothesis in the context of complex social-ecological systems—a system wherein human and environmental dimensions are interdependent, where common-pool resources are used or managed by multiple types of stakeholders. Using a fisheries example, we examine the relationship between resource stakeholders’ identities and their cognitive diversity. We used cognitive mapping techniques in conjunction with network analysis to measure cognitive distances within and between stakeholders of various social types (i.e., identities). Our results empirically show that groups with higher identity diversity also demonstrate more cognitive diversity, evidenced by disparate characteristics of their cognitive maps that represent their understanding of fishery dynamics. These findings have important implications for sustainable management of common-pool resources, where the inclusion of diverse stakeholders is routine, while our study shows it may also achieve higher cognitive coverage that can potentially lead to more complete, accurate, and innovative understanding of complex resource dynamics.

## Introduction

Diversity is a term generally used to identify differences between individuals or describe instances of being composed of differing elements or including different qualities. Depending on the type of differences by which the diversity is determined, people can be categorized under demographic, cultural, political, occupational, intellectual, or many other categories. As a guiding principle, these dimensions can be dichotomized into two overarching kinds of diversity: (a) *identity diversity* and (b) *cognitive diversity* [1–3].

Identity diversity—also known as *surface-level* diversity—refers to differences in a set of subjective characteristics that are apparent across individuals or groups [2]. As such, many social categories or deductive specifications that are explicitly defined by demographic, socioeconomic, cultural, political, or any other salient features of the individuals fall into identity diversity. These are factors that are generally considered observable (think demographic categories), and often perceptible by those who seek or care about diversity and inclusion [4].

On the other hand, cognitive diversity—also known as *deep-level* diversity—refers to differences in how people represent, think about, and solve problems [2]. Hong and Page (2004) refer to this kind of diversity as *functional differences* and explain how it might be determined by measuring variations in people’s perspectives (i.e., how they represent a problem) and heuristics (i.e. how they find solutions to a problem) (also see [5]). This kind of diversity has been suggested to be a critical driver of improving group performance in complex problem-solving [6–8]. Three mostly cited problem-solving benefits associated with cognitive diversity are i) augmentation (i.e., the generation of a larger pool of knowledge), ii) purification (i.e., the cancelation and refinement of errors and inaccuracies mostly in predictions), and iii) recombination (i.e., the emergence of innovative solutions as a result of higher possibility for permutation and combination of knowledge) [6, 9–11]. Accordingly, since human societies face more complex problems today, cognitive diversity becomes a vital ingredient in contemporary problem-solving.

Despite these benefits, achieving cognitive diversity is not always straightforward because such differences across groups and individuals are not immediately observable or readily detectable [12]. Instead, to assess cognitive diversity, researchers need to dive deeper into invisible variations in personality, intellectual abilities, and cognitive characteristics of individuals using intelligence tests [13], psychological and neuropsychological assessments [14], mental modeling techniques [15], or cognitive ability tests [16]. Yet, seeking and embracing cognitive diversity does not necessarily satisfy the full inclusion of diverse social identities [2, 17], which can be problematic wherein social inclusion is vital to achieving ethical goals such as achieving social equity and resolving conflicts in areas like participatory governance [18].

However, the inverse may possibly be true—that is, some sort of identity diversity can congruently achieve beneficial cognitive diversity [19, 20]. Under certain circumstances, incorporating diverse identities into problem-solving may concurrently encourage cognitive diversity which is beneficial to groups’ problem-solving capability, while it also satisfies the social equity goals. While it does not appear to be an unreasonable assumption in some cases (e.g., particularly those cases wherein some salient differences that determine identity diversity are of high problem-solving relevance) [21], the literature around “diversity” is still open to debate about the relationship between identity and cognitive diversity. In fact there is evidence to the contrary, that is, that identity diversity does not always contribute to beneficial cognitive diversity [2, 3, 17].

Regardless of these controversies, in many cases, achieving both kinds of diversity at the same time or what has been referred to as “congruence between surface and deep-level characteristics”—has been thought to be a major success [2]. Despite practical challenges, implementing this congruency has been recommended in multi-stakeholder governance and management systems such as common-pool resources and environmental assessments [18]. Understanding diversity within stakeholders who interact with natural resource systems, therefore, serves as a great case study to evaluate these congruencies since they typically involve multiple stakeholders and require the participation of socially diverse groups in dialogue, deliberation, decision-making, and adaptive co-management [22]. This inclusive participation of socially diverse groups of stakeholders in decision-making and policy development processes instills stakeholders’ sense of ownership of the decisions, helps them address conflicts and build shared understandings, thereby improving the legitimacy of natural resource management strategies [23, 24]. It, thus, constitutes an important component of improving decision-making and social and environmental sustainability [25].

In addition, natural resource systems are composed of both social components (i.e., human-related factors like consumption, regulations and conservation) and ecological components (i.e., nature-related factors like ecosystem health, resource abundance, productivity), as well as their feedback interactions (e.g., the impact of consumptions or regulations on resource dynamics or the impact of degraded ecosystem productivity on human well-being). These so-called social-ecological interdependences commonly lead to complex system behaviors and dynamics that are hard to predict [26, 27]. As a result, understanding and managing natural resource systems typically entails the participation of cognitively diverse individuals who bring a wider range of perspectives and heuristics to the table, and their diverse knowledge pool can lead to a greater cognitive coverage and a boosted problem-solving capability [7, 8, 28, 29].

Here we explore whether *congruence* exist in groups with diverse environmental stakeholders who self-identify themselves in different professional roles, each represents a certain type of human-nature interactions. We hypothesized that these different social identities are associated with distinct cognitive spaces and knowledge (i.e. there exist a correlation between surface and deep-level diversities in environmental stakeholders).

We build this hypothesis on prior theoretical and empirical evidence describing that different social groups of resource stakeholders (e.g. fishermen, hunters, scientists, policymakers, and managers) interact differently with natural and social dimensions of ecosystems at different time and spatial scales. Such different social groups may also be subjected to diverging beliefs and values [30], disparate experiences with the nature [28], differences in preferred adaptation strategies and management policies [31], and are thought to build in their minds diverse cognitive representations (i.e., mental models) of the system that reflect their specific interests and interactions.

To empirically support our hypothesis, we use a fisheries example where multiple groups of stakeholders interact differently with a natural ecosystem (i.e., a common-pool resource system). Our case is the Western Baltic cod (*Gadus morhua*) in Germany. Western Baltic cod is of crucial importance for regional ecosystems and constitute a vital component of coastal economies [32, 33] (a more detailed explanation of the case study is provided in S1 File). Cod is known as one of the species in high demand and plays a key role in the Baltic Sea, environmentally, socially and economically [34]. Here, we focus on stakeholder groups who are differently affected by or involved in fisheries management and therefore represent varying interdependences with the natural ecosystem (S1 File).

We use a semi-quantitative cognitive mapping technique called Fuzzy Cognitive Mapping (FCM) [35, 36] in conjunction with network analysis to develop a novel approach to measuring cognitive distances within and between social groups of stakeholders (i.e. individuals with diverse roles and resource use). Finally, analyzing the congruence of differences in stakeholders’ identities and features of their cognitive maps can empirically demonstrate the potential proximity of surface and deep-level diversities among environmental stakeholders.

## Methods

### Data collection

#### Mental models and fuzzy cognitive maps

To measure variation in stakeholders’ perceptions and understanding of the complex social- ecological relationships (i.e. deep-level diversity), we collected individual mental models about fisheries ecosystem dynamics and management from fisheries stakeholders. Theoreticians have hypothesized that humans develop in their mind simplified internal representations of the complex reality that allow them to perceive the world around them [15]. Individuals who observe, interact with, and experience the world around them can concurrently develop an internal model of the external world to understand it and predict how it functions [37]. These so-called mental models represent patterns of perceived cause-and-effect relationships among various concepts that are built through reasoning and thus shape the basis for problem-solving and decision-making [38]. Importantly, these mental models can be elicited through cognitive mapping techniques [39]. Cognitive maps are graphical representations of mental models in the form of directed networks where nodes represent concepts and edges show the causal relationships between them (see S1 File for more details).

Here we used Fuzzy Cognitive Mapping (FCM) [35]—an enhanced form of cognitive maps which mathematically and graphically model system components (nodes), their causal relationships (edges), and the strength of these relationships using a normalized quantitative parameterization of causal magnitudes. In an FCM, edges are characterized by a normalized number in the interval of [-1, +1], corresponding to the strength and sign of causal relationships between nodes, thereby forming a weighted directed graph [40]. These weighted directed graphs can be analyzed using network analysis through measures and algorithms related to node connectivity, graph distances, their adjacency matrices similarity, and graph clustering [41].

#### Cognitive map elicitation

Five relevant stakeholder types were identified in a stakeholder analysis: Local fisheries (including commercial and recreational fishers) (33.3%), representatives of tourism industry (12.1%), Non-governmental organizations (NGOs) (18.2%), managers and policymakers (18.2%), and scientific experts (18.2%). Two key criteria were applied to sample study participants (N = 33) using a purposeful sampling strategy: stakeholders needed to be affiliated with a German institution either through their job or honorary position, and have been active (involved or affected) in the cod fishery in the Western Baltic Sea for more than 5 years (see the description of interviewed stakeholders in S1 File). The first criterion is based on the intention of a national survey, whereas the second one was chosen as a reference point to ensure that the interviewees have established themselves in their position (job, volunteer) and are familiar with the subject of cod fishery in the Western Baltic Sea. Both criteria led to the exclusion of some actors, including stakeholders from the fishing industry or people who have only recently started working on this topic, for example, trainees.

We elicited stakeholders’ FCMs through semi-structured interview processes. This study was conducted with approval of University of Hamburg, and informed consent was acquired from all participants. All subjects gave their informed consent via email for inclusion before they participated in the study. The study was hence conducted in accordance with the Declaration of Helsinki. Individuals were asked to identify relevant concepts (i.e., system components) and their causal relationships, from which they then drew a concept map representing their mental models about Western Baltic cod ecosystem and fisheries management. This process included routine FCM data collection practices with open-ended concepts [42]. Participants’ cognitive maps were qualitatively homogenized (i.e., using the same terminology for concepts that have the same meaning across all individual maps; see refs.[43, 44] for more detail about qualitative homogenization and standardization process) and digitized after the interview (i.e. maps were converted to digital weighted directed graphs and corresponding adjacency matrices using www.mentalmodeler.org) and sent back to the interviewees for validation. We described in details the cognitive map elicitation protocol elsewhere [45] and in S1 File.

### Data analysis

#### Comparing graphs

Following the qualitative homogenization of FCMs and standardization of terminologies used to describe their concepts, we conducted a subsequent level of homogenization called quantitative homogenization: FCM adjacency matrices were brought to the same size and thus included information about every unique concept that was mentioned in any of the contributing FCMs. By doing this, all adjacency matrices were adjusted to have the same size in favor of matrix comparability—for each individual FCM, the absent nodes not mentioned in the original map were added but left unconnected to other nodes.

To measure cognitive diversity in a group, we determine how dissimilar the cognitive maps of the group members are by measuring the average of their pairwise distances. To quantify the distance between cognitive maps, we perform network comparisons of FCMs. Each FCM is a directed, weighted graph *G*(*V*,*E*), with *V* being the set of nodes (i.e. set of homogenized concepts mentioned by all individuals) and *E* being the set of edges (i.e., causal connections). We compute the distance between a pair of FCMs by taking into account two measures:

- *The distance between the dichotomized adjacency matrices of their graphs*:

The dichotomized adjacency matrix *A*^*d*^ of a graph *G* is a *n* × *n* square matrix, where *n* is the number of nodes, and the elements of the matrix [*a*_*ij*_] indicate whether pairs of nodes *i* and *j* are adjacent [*a*_*ij*_] = 1 or not [*a*_*ij*_] = 0 in the graph. Apart from weightings, in FCMs, the presence and absence of the connections is important information which is a binomial variable (0 or 1), representing the extent to which one individual includes or excludes the directed causal relationship between two concepts when representing a complex system (independent of the sign and the strength of the relationships). One common norm used as graph distance is the Jaccard distance [46]. Given two graphs *G*_1_(*V*_1_,*E*_1_) and *G*_2_(*V*_2_,*E*_2_) with dichotomized adjacency matrices $A_{1}^{d}$ and $A_{2}^{d}$, the Jaccard coefficient *J* is defined as $J(A_{1}^{d},A_{2}^{d})=\frac{A_{1}^{d}\cap A_{2}^{d}}{A_{1}^{d}\cup A_{2}^{d}}$, and their Jaccard distance is calculated as follows:

$$d_{J}=1-J(A_{1}^{d},A_{2}^{d})$$ (1)

- *The distance between the spectra of their graphs*:

The spectrum of a graph *G*(*V*,*E*) is the set of eigenvalues of its normalized Laplacian [47, 48] and contains useful information about the principal properties and structure of a graph which has important implications for graph comparisons [48–50]. In addition, the prior study [25] demonstrated that the Euclidian distance between the spectra of two FCMs perfectly matches the distance between dynamics of causal relationships as perceived by individuals (i.e. simulation of what-if scenarios using a combination of fuzzy logic and artificial neural networks) [35, 51].

Let *A*_*w*_ be the undirected, weighted adjacency matrix such that the elements of *A*_*w*_ = [*a*_*ij*_] = [*a*_*ji*_] indicate the edge weights between pairs of nodes *i* and *j* that are adjacent in the graph. Then the (symmetric) normalized Laplacian is defined as *L*^*sym*^ = *D*^-1/2^*LD*^-1/2^, where *L* = *D*—*A*_*w*_, while *D* is the degree matrix. Importantly, all eigenvalues of the normalized Laplacian are real and non-negative [48], thereby offering a practical tool for measuring graph distances. Given two graphs *G*_1_(*V*_1_,*E*_1_) and *G*_2_(*V*_2_,*E*_2_) we find a set of all eigenvalues for each normalized Laplacian as their spectra. Similar to the approach outlined in [52, 53], we compute the Euclidian distance between the graphs’ spectra *d*_*s*_ as follows:

$$d_{s}=\sum_{i=1}^{k^{*}}{\left(\lambda_{1i}-\lambda_{2i}\right)}^{2}$$ (2)

where *λ*_*i*_ is the *i*^*th*^ largest eigenvalue and (*λ*_*i*_ ≥ 0 *for* ∀ *i*). We find the smallest *k* such that the sum of the *k* largest eigenvalues constitutes at least 90% of the sum of all of the eigenvalues. If the values of *k* are different between the two graphs, we use the smaller one k*.

These two measures of graph distance are complementary as they take into account the structural properties that are characterized by either edge directionality or edge weights. Thus, to jointly acknowledge the weight and directionality of causal connections in FCMs, we define the cognitive distance between two FCMs as follows:

CD=ds1−dJ×φ (3)

were *φ* is the standardization coefficient for mapping *CD* to a normalized range between [0,1].

All individual cognitive maps were converted into adjacency matrices and the cognitive distances between any pairs of maps were computed using Eq 3. For each identifiable social group (e.g., fishers, managers, NGOs, tourism, and experts) we make two sets of cognitive distances: the *intra-group* set including the cognitive distances between any pairs of socially homogeneous individuals who share the same social category (e.g., a pair of fishers), and the *inter-group* set including the cognitive distances between any pairs of socially diverse individuals who do not share the same social category (e.g., a pair of one fisher and one manager). Independent sample t-tests were used to compare the means of cognitive distances in intra-group and inter-group sets. This helped us determine whether or not inter-group distances were longer than intra-group distances—that is, the cognitive diversity amongst socially diverse individuals was statistically significantly higher than the cognitive diversity in socially homogeneous ones. Despite the fact that independent-samples t tests were shown to be reasonably robust to Type I and Type II errors when the normality assumption was violated [54], we conduct an additional non-parametric test to determine the significance of differences. We use the non-parametric Wilcoxon-Mann-Whitney (or *U*) test to compare differences between *intra-group* and *inter-group* sets of cognitive distances with the assumption that they are independent, but not normally distributed.

#### Monte-Carlo method

We then used the Monte-Carlo method (MCM), wherein the virtual FCMs were randomly reproduced from the probability distributions of stakeholder-driven cognitive maps. That is, virtual agents with a defined identity (e.g., fisher, manager, etc.) were computationally generated, such that their cognitive maps were randomly drawn from the probability distribution of FCMs elicited from actual individuals of that social type [8]. For each group *g* with *K* individuals *i* = 1,…,*k*, the set of all unique edges mentioned by these individuals is {*E*}:

$$\{E\}=\cup_{i=1}^{k}\left\{E^{i}\right\}$$ (4)

$$for\;\forall \;e\in \{E\},\;\pi_{e}=\frac{1}{K}\sum_{i=1}^{K}X_{e}^{i}$$ (5)

Where *π*_*e*_ is the frequency of edge *e* in group *g*, {*E*^*i*^} is the set of edges included in the FCM of individual *i*, and $X_{e}^{i}=1$ if *e* is in {*E*^*i*^}, ($X_{e}^{i}=0$, otherwise). Then, a random FCM is generated in two steps: First, a random set of edges is drawn such that the probability that the generated FCM includes edge *e* is determined by a Bernoulli distribution, *Pr*(*X*_*e*_ = 1)∼*Bern*(*π*_*e*_); and second, the weight of edge *e* in a random FCM *w*(*e*) is determined by a random normal distribution:

$$w(e)∼N(\mu_{e},\sigma_{e})$$ (6)

Where *μ*_*e*_ and *σ*_*e*_ are the mean and standard deviation of weights assigned to edge *e* by all individuals in group *g* whose FCMs include *e*. Although, this process of random FCM generation uses edges-probability distribution (instead of nodes-probability distribution), which represents the likelihood that two-nodes co-occur, and at the same time, they are adjacent, it does not take into account the probability that two edges with a shared source node co-occur in a map. One possible solution to this limitation is to keep at least a memory order of one and hence using a Markov-Chain Monte-Carlo (MCMC) of memory 1 in which the random matrix is reproduced while representing a memory with respect to the number of first neighbors that each node has. However, this requires a relatively large sample of observations (i.e., collected FCMs), which in many cases, is not achievable due to the fact that FCM interviews are typically time and resource demanding.

Importantly, the MCM helps us regenerate virtual samples of stakeholders that artificially represent various levels of identity diversity, thereby enabling us to carry out a probabilistic examination of how identity diversity correlates with cognitive diversity. Using MCM we built 100 replicates of our FCM sample. Each reproduced sample has *N* = 33 individuals (to resemble actual sample size) with a random combination of virtual agents from different social categories (i.e. individuals of different types). For each random replicate, 1000 bootstrap resamples were used to estimate the 95% confidence interval.

**Fig 1:**
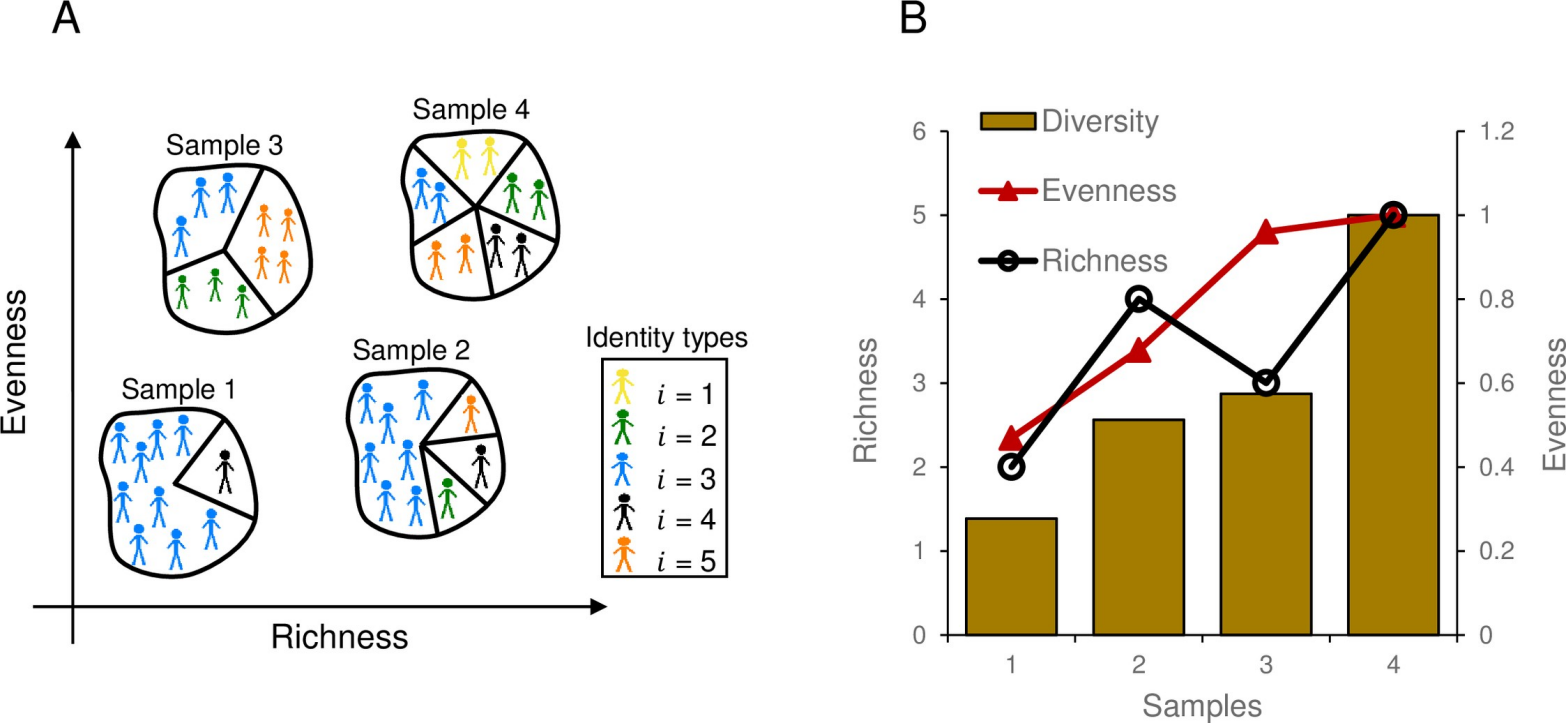
Illustrative samples of individuals with different levels of richness and evenness. Four hypothetical examples with low-to-high levels of richness and evenness are shown in (A). The calculated identity diversity with regards to each sample’s richness, evenness, and their influence on the level of diversity is shown in (B). Samples’ diversity was calculated using an information theoretic measure built on Shannon’s entropy formula.


To measure the identity diversity of each reproduced sample we used Shannon’s entropy index (*H*) [55]. The Shannon’s entropy index takes into account both the richness (i.e., how many unique identities exist in a sample) and the evenness (i.e., how even the proportions of stakeholder identities are in a sample), and thus provides useful information about identity diversity. Fig 1 displays four illustrative samples of size 10 with different richness and evenness. We calculate identity diversity in each sample using the following equations:

$$H=-\sum_{i}p_{i}\times Ln(p_{i}),\;p_{i}=\frac{n_{i}}{N}$$ (7)

$$D=\frac{e^{H}}{\text{max}\;(r)}$$ (8)

where *H* is the Shannon’s entropy index, *n*_*i*_ is the number of individuals of type *i*, *N* is the sample size, *D* is the identity diversity, and *max*(*r*) is the maximum possible richness (i.e. maximum possible number of unique types) in a sample, which is 5 in our case. *D* is a number between [0,1] with values closer to one representing higher diversity. In addition, we define cognitive diversity as the mean of pairwise cognitive distances (*CD*) (see Eq 3) between any two individuals within the sample. Finally, the correlation between identity diversity and cognitive diversity is calculated using Pearson correlation coefficient.

**Fig 2:**
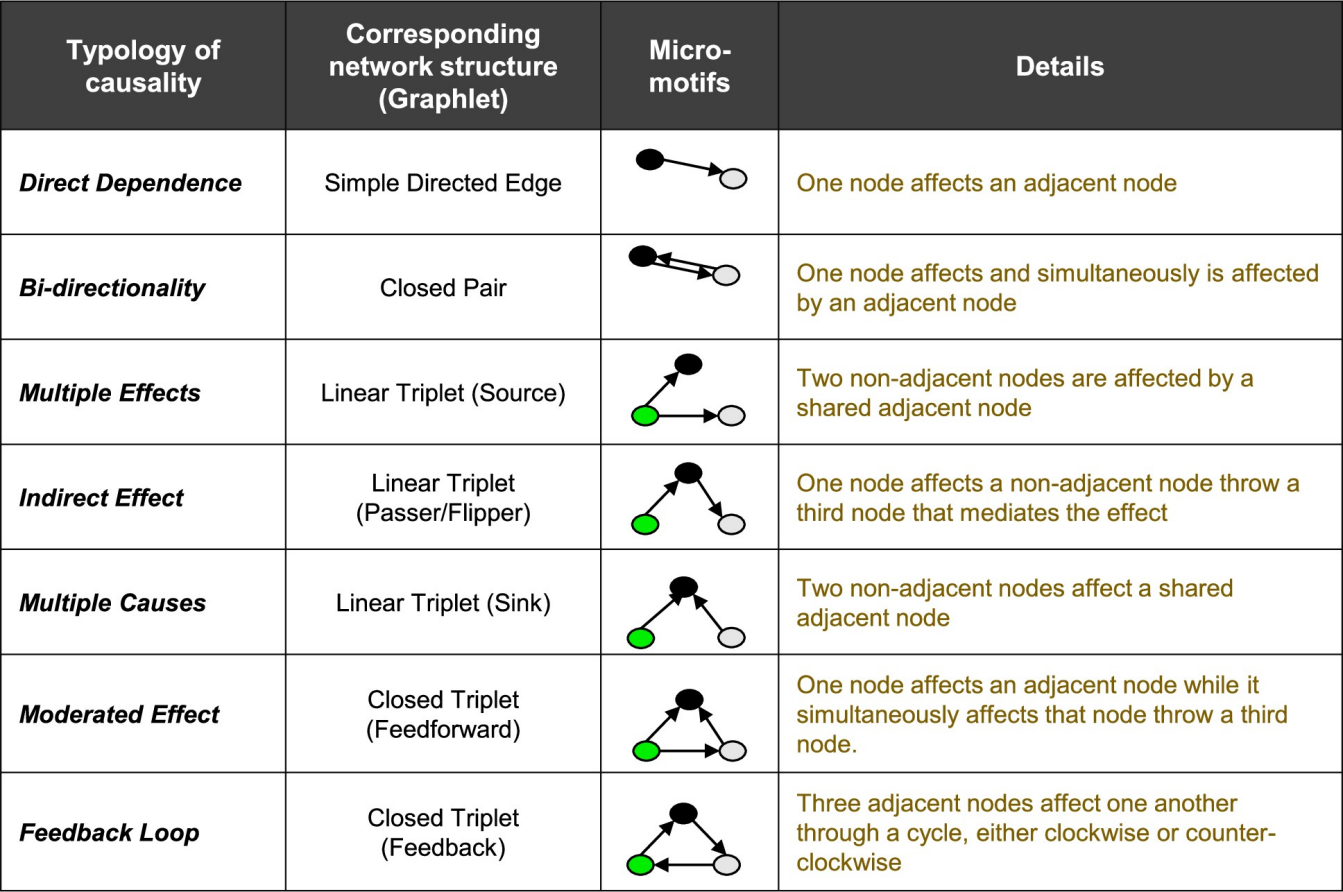
Seven micro-motifs and their corresponding network structure (Graphlet). These micro-motifs exemplifying common patterns of perceived causation in cognitive maps.

Last but not least, we drew on network theory and cognitive map analyses of perceived causation [56] to cluster FCMs using their network micro-motifs (i.e., micro-structures that are constructed by two or three nodes and some unique patterns of connections between them, which shape the underlying elements of perceived causation in a cognitive map). The frequency distribution of these micro motifs in one cognitive map—also known as directed graphlets of size two and three—can provide useful information about how one individual sees the causal interdependencies and can be used as a tool for deep-level comparisons [51]. Theoretical and empirical studies have frequently suggested the use of seven simple micro-motifs (Fig 2) to exemplify common patterns of perceived causation [56–62]. We combined Principle Component Analysis (PCA) and K-mean clustering to develop an unsupervised learning algorithm that clusters individuals based on their frequency distributions of these 7 micro-motifs and no pre-defined labeling. We also clustered the individuals based on their pre-defined identities (i.e. social types labeling). Analyzing and visualizing the alignment between identity-based clustering and micro-motif-based clustering helped us further examine the proximity of surface and deep level diversities.

## Results

### Intra versus inter-group cognitive distances

**Fig 3:**
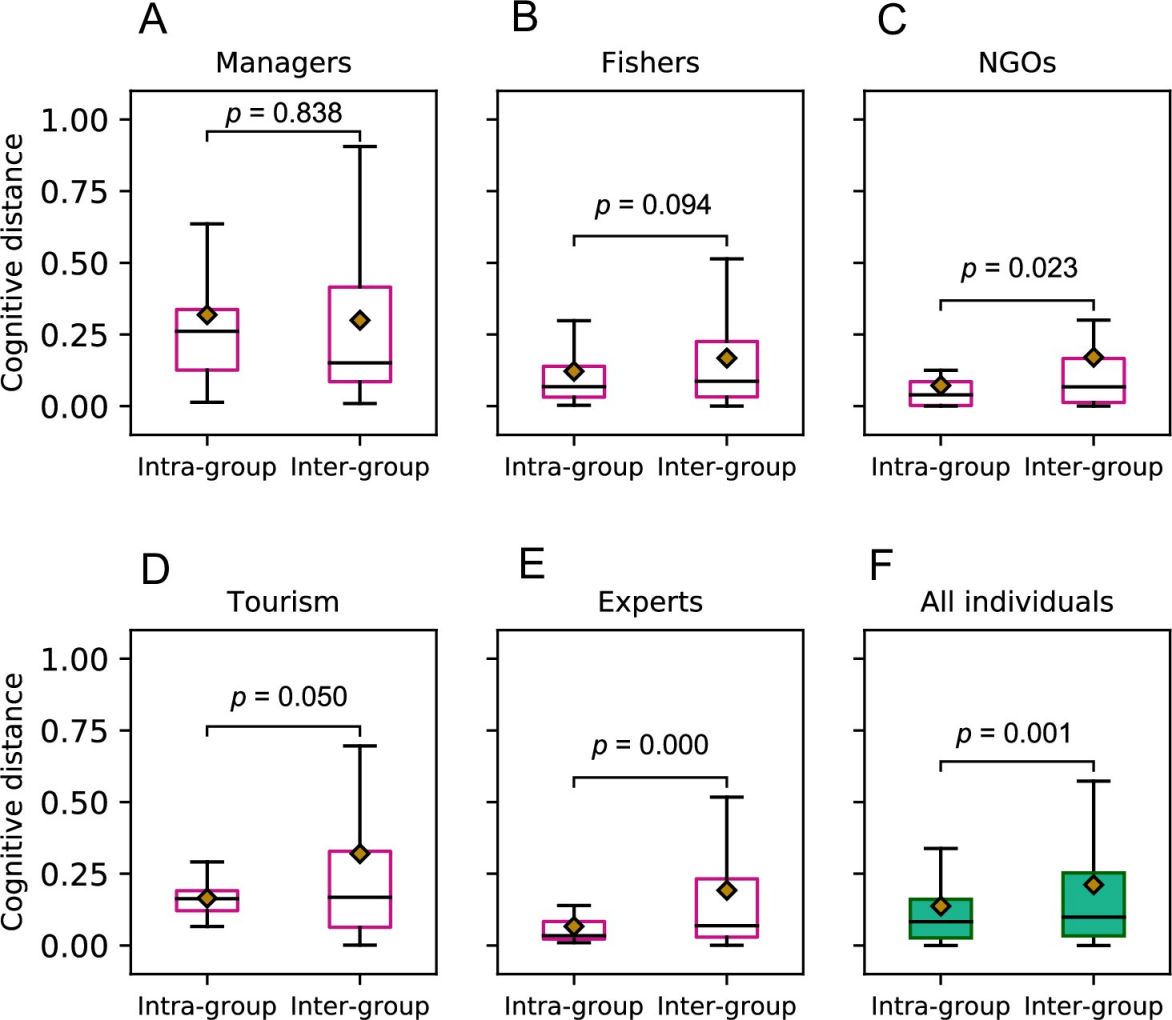
Comparison of intra-group (i.e., within social groups of stakeholders) versus inter-group (i.e., between social groups of stakeholders) pairwise cognitive distances. Independent sample t-tests were used to compare the means, and *p*-values demonstrate significance of their difference. (A-E) show the distribution of inter-group versus intra-group cognitive distances for five social groups of stakeholders. Overall results for all individuals is shown in (F). Note that in (F) the nonparametric Wilcoxon-Mann-Whitney *U* test is also significant (*p* = 0.04).

We collected 33 FCMs through semi-structured interviews with stakeholders (see S1 File). Five social groups (i.e. types) of stakeholders participated in our study. Fig 3 illustrates the comparison of intra-group versus inter-group pairwise cognitive distances (see Eq 3). Independent sample t-tests were used to compare the means, and p-values demonstrate significance of their difference. In all five socially distinguishable groups of stakeholders (Fig 3A–3E) the mean of inter-group cognitive distances is longer than the mean of intra-group cognitive distances, and in three groups (i.e., NGOs, tourism, and experts) these differences are statistically significant at the level of *p* < 0.05. It is visible from the Fig 3F that, once all individuals are combined, the cognitive diversity (measured by the mean of cognitive distance between any pairs of individuals) amongst socially diverse individuals (i.e. inter-group pairs) is statistically significantly higher than the cognitive diversity in socially homogeneous ones (i.e., intra-group pairs). Additionally, the results of nonparametric Wilcoxon-Mann-Whitney *U* test further supported the findings that the mean of inter-group cognitive distances was statistically significantly larger than the mean of intra-group distances (*p* = 0.04), even if the the normality assumptions were violated.

### Correlation of identity and cognitive diversity

**Fig 4:**
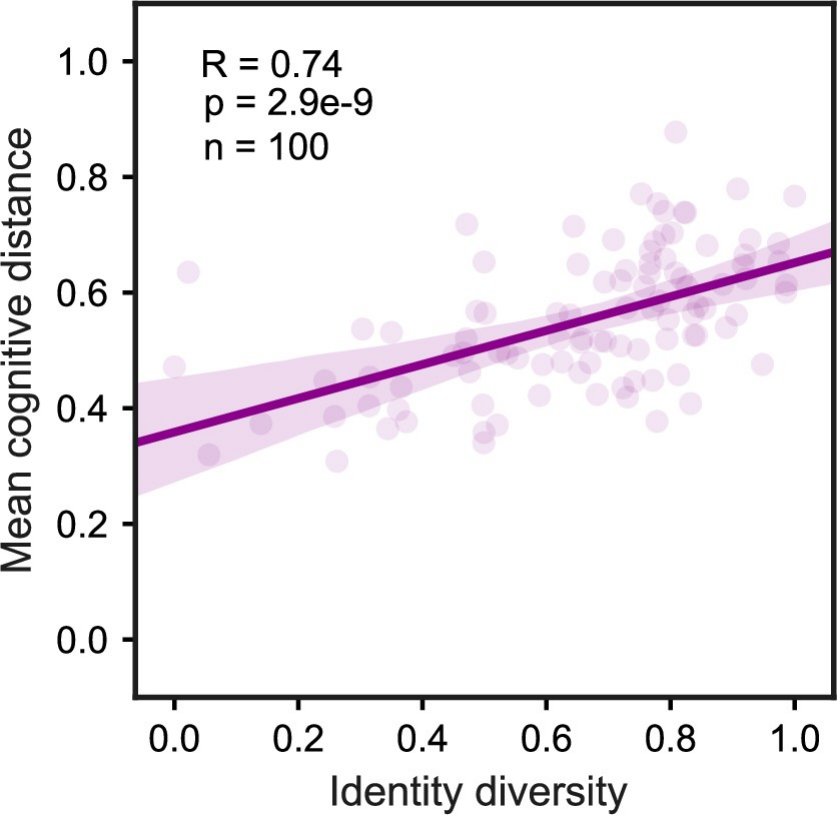
Correlation between identity and cognitive diversity. The figure shows the result of 100 randomly generated samples of size N = 33 individuals. The shaded area represents 95% confidence interval estimated by bootstrapping the sample 1000 times.

Next, we examined the correlation of identity and cognitive diversity using the MCM. Fig 4 shows the result of 100 randomly generated samples of stochastic agents (i.e. artificial individuals who own randomly-generated cognitive maps drawn from the probability distribution of actual FCMs). These random samples represent different levels of identity diversity determined by Eq 8. Pearson correlation coefficient of 0.74 revealed a positive association between samples’ identity diversity and the mean of pairwise cognitive distances among agents’ cognitive maps. That is, samples high in identity diversity are 74% probable to show high cognitive diversity (each sample was bootstrapped 1,000 times to estimate 95% confidence interval).

### Proximity of surface and deep-level clusters

**Fig 5:**
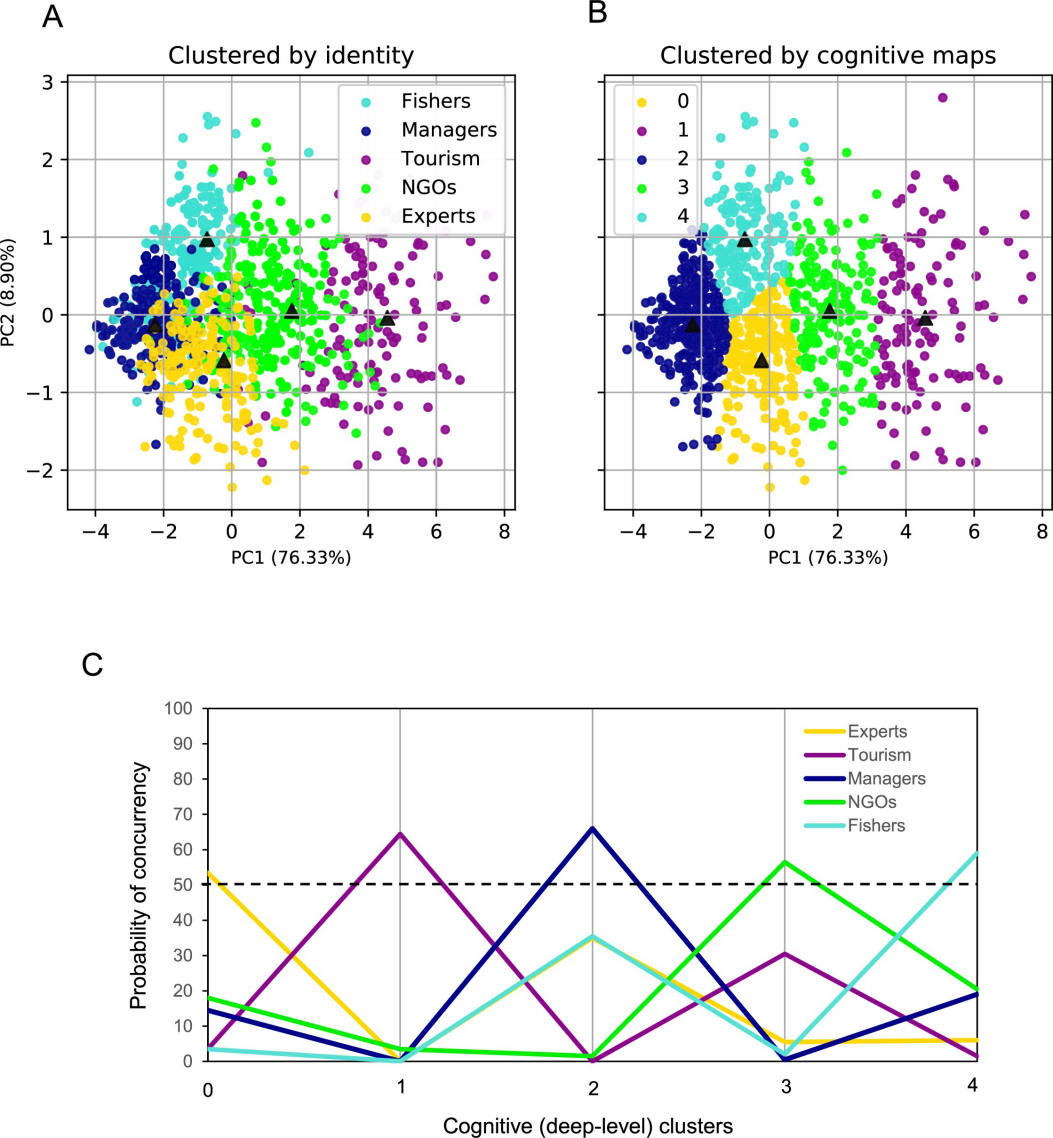
The proximity of identity and cognitive diversities based on micro-motifs in cognitive maps. Principle component analysis was performed on the seven dimensions of micro-motif frequencies in 1000 random cognitive maps (with 200 individual maps were re-produced form the probability distribution of cognitive maps of each of the five social groups). Two principle components were retained, cumulatively explaining about 85% of variance. Individual maps are illustrated by points in a 2-dimensional principle component scatter plot where points are clustered by their predefined social identities in (A) and by a K-Mean clustering algorithm using the Euclidian distances between points in (B). Black triangles in (A) and (B) illustrate the center of the clusters based on K-nearest neighborhood. The probability of concurrencies of social identities and cognitive clusters is shown in (C).

In addition, we compared the results of two clustering algorisms: one based on predefined socially distinguishable labels (i.e. identity), and the other one based on an unsupervised dimension reduction technique (i.e., a PCA) that transforms cognitive maps from their 7-dimensional micro-motif space (see Fig 2) to a 2-dimensional principle component space, where clusters are determined by k-nearest neighbors (based on their Euclidian distance). Fig 4A and 4B illustrate the results of these two clustering algorithms for a randomly reproduced sample of size 1,000 (with 200 individuals for each of five social groups). It is visible from these figures that individuals who are similarly clustered by their predefined social identities are more likely to be in the same cognitive neighborhood that represents a prevailing cluster of individuals who are closely matching in terms of how they perceive causal interdependences. Fig 5C shows the probability of possible concurrencies formed by the categories of two clustering algorithms. Interestingly, for each cognitive cluster, there exists one and only one dominating social cluster (i.e. identity) whose concurrency probability is greater than 0.5, meaning that the overwhelming majority of individuals within a cognitive cluster share the same social identity. These findings revealed that environmental stakeholders demonstrate distinguishable differences in discrete aspects of their cognitive models (i.e., deep-level clusters) that are most probably aligned with the way they could have been distinguished by their disparate social identities (i.e., surface-level clusters). Consequently, these perfect alignments demonstrate the strong likelihood of congruence of surface and deep level diversities in environmental stakeholders.

## Discussion

The importance of diversity, in general can be seen across systems, from ecosystems [63] to economic systems [64], and also extends to norms regarding social inclusion and social equity [65, 66]. In each case, diversity is considered to make systems more adaptable and resilient to changes. Here we extend this general notion of the diversity bonus [4] and provide evidence of the hypothesized correlation between identity diversity (surface) and cognitive diversity (deep). Our data provide empirical evidence that the inclusion of diverse stakeholder groups in natural resource problem-solving has implications for better understanding the complexity of natural resource systems since different social groups interact within these systems somewhat similarly by group, but distinct across groups providing more opportunities for full cognitive coverage [8]. While the literature on collaborative natural resource management has for some time promoted the inclusion of diverse stakeholder groups and public participation for improved decision-making [18], the diversity of knowledge systems that these different social groups bring with them has been largely assumed, rather than empirically evaluated with some exceptions [67].

Combining approaches from network science, graph theory, and cognitive mapping, we explored the relationship between social identity and cognitive diversity in environmental stakeholders who interact with a common pool resource system. For this, we collected stakeholders’ cognitive maps using FCM—a weighted, directed graph that visualizes people’s mental models that represents how each individual perceives causal interdependencies to explain the complex real world they interact with. In this study we used a case of Western Baltic cod in Germany and collected FCMs from five groups of stakeholders whose identities are socially distinguishable (i.e., social categories that are identified by their distinct roles and types of resource use which specify how they interact with the fisheries ecosystem and its resources).

Our data indicate that individuals whose identities (i.e., social categories) vary and demonstrate variations at surface-level, also develop cognitive maps that are more likely to demonstrate diverging network structural aspects as determined by their longer cognitive distances—a quantitative measure to represent cognitive, deep-level variations base on cognitive map characteristics. We developed a novel measure of cognitive distance which simultaneously takes into account dissimilarities in graph and spectral-graph metrics to provide useful insights about how individuals’ cognitive maps (i.e., mental models) differ at the macro scale (metrics that represent a graph as a whole). These methods for comparing FCMs prove to produce useful information about how individuals represent different mental models and how they vary in perceiving the dynamics of the system they represent (e.g. [8, 25]).

Despite the benefits of using spectral methods such as the eigenvalue similarity index we used in the current study, we should warn the readers of some of the drawbacks associated with the use of spectral methods in comparing graphs (e.g., dependence on the matrix representation and abnormal sensitivity, such that small changes in the graph’s structure can produce large changes in the spectrum) [51]. Thus, before using this approach, we encourage readers to evaluate the sensitivity of the spectra of their observed sample of FCMs to small changes (e.g. through a repetitive process of random small alterations, such as random removal or extension of nodes and edges). We, also encourage readers to replicate our study by using other methods for comparing graphs and conclude which approaches most appropriately fit their study.

Yet, it is also interesting to consider the variations of cognitive maps at the micro scale. To that end, we examined the distribution of certain directed graphlets in cognitive maps (i.e. micro-motifs) that represent common patterns of perceived causalities and are building blocks of causal reasoning [56]. This micro-motif comparisons, too, showed the proximity of identity and cognitive diversities (Fig 5). While conventional graphlet methods for network comparisons such as Relative Graphlet Frequency distance (RGF-distance) [68] or Graphlet Degree Distribution agreement (GDD-agreement) [69] use all 2-5-node Graphlets, our approach only takes into account those micro-motifs that represent common typology of perceived causation and have important relevance to comparing cognitive maps (see Fig 2).

### Trade-offs in measuring knowledge diversity

Currently, there are several methods that exist to elicit and compare knowledge diversity, each with trade-offs. For example, “Cultural Consensus” theory [70] is a relatively straightforward way to understanding within and across group differences, often measured through evaluating individuals’ responses to a series of related questions where norms and shared beliefs can be assessed through aggregate responses [71]. While these methods have been widely used, many questions posed to individuals exist at a broad-level and often force participants to select binary responses (true/false). Additionally, qualitative approaches, such as applying emergent coding rubrics to concept maps or narratives are also common [72]. While these approaches provide rich data, analyzing and coding qualitative concept maps take considerable time and are resources that might not be available with larger datasets. Finally, FCM as a semi-quantitative assessment, such as the approach we use here, has been popular in recent years. Gray et al. (2014) point out, however, that there are considerable trade-offs in how these cognitive maps are collected: are concepts/elements in the model pre-defined?, are these maps the result of an interviewer leading the process of map development or are crowdsourced freely, or are they a mix of data collection methods? Each decision a researcher makes in the data collection process will influence the analytical options available to the researchers and should be considered fully in the design of studies seeking to elicit, capture and integrate or compare individual knowledge [42].

In the context of social-ecological systems, and in contrast to our findings, Stier et al. (2017) found that experts can exhibit cognitively diverse views and perceptions about the structure of a complex ecosystem (e.g., marine food web), independent of commonly identified “bins” of expertise (e.g., local, scientific, traditional) [17]. That is, the identity and cognitive diversities may not necessarily co-occur. The authors of that study have contended that individuals’ demographics and background may not explain differences in perceptions of complex ecosystem structure as evidenced by lack of variations in their cognitive maps.

We argue that such findings might be influenced by the methodological biases resulting from highly standardized elicitation methods where cognitive maps are constructed using pre-defined standardized concepts provided by researchers. In such cases, representation of individuals’ cognitive maps is significantly influenced by researchers’ presumptions or limitations; consequently, true cognitive diversity is less likely to be fully captured. Therefore, we decided to provide more flexibility and elicited cognitive maps while individuals were able to freely brainstorm, represent concepts, and draw connections between them with no influence from researchers and facilitators. This decentralized process allows individuals to freely represent their internal perceptions and system knowledge, and therefore it increases the probability that a wider spectrum of knowledge diversity (i.e., cognitive coverage) is sampled. In addition, conventional methods to compare FCMs (e.g. methods described in [51]) which were used by prior studies (e.g., [17]), take into account fewer structural metrics mainly obtained by comparing the value of network global statistics, such as the density, number of receiver/driver/ordinary nodes, complexity index, hierarchy index, and the centrality of particular nodes. Except for the centrality, these metrics do not consider the correspondence between nodes—that is, two FCMs with different set of nodes, (i.e., different qualitative compositions) may be considered very similar only because they have the same number of nodes or how these nodes are connected to each other matches across two FCMs (i.e., apples and oranges considered similar because they both have round shapes). These limitations may impact the results of previous studies. Here we addressed these limitations by introducing a novel approach to measuring cognitive distances within and between groups of stakeholders.

## Conclusion

In sum, our approach produces a more inclusive set of insights into understanding and measuring within group and between group knowledge variations, which has three important implications: First, measuring within group cognitive distances has implications for how we understand similarities and knowledge homogeneity within "social groups", which enables innovative approaches to measuring culture (shared ideas and knowledge) and group-specific cognitive biases or alternatively, identifying different types of expertise (e.g. commercial fishermen may have more expertise about biological or market-related aspects of a fishery compared to other groups). As our study supports, individuals form the same social group hold more similar knowledge, and this might be attributed to their shared experiences, beliefs and values; the routine set of human-environment interactions they adopt in their day-to-day life; and a more frequent exposure to the same information sources and social network (e.g., shared media and news outlets). They, therefore, build in their minds cognitively more homogenous understanding of the complex ecosystem dynamics compared to the members of other social groups. Our novel approach of measuring within group shared knowledge helps us to understand how different social groups construct their specific cultural spaces about the environment which, in turn, lead stakeholders to behave/adapt in a certain way in response to environmental and social changes.

Second, measuring across group cognitive distances has implications for understanding how incorporating diverse knowledge and perceptions from across groups may ensure problem-adequate solutions, reaching knowledge saturation points, and the achievement of more complete “cognitive coverage”. Knowledge held by stakeholders varies across social groups, yet suggesting that different types of stakeholders hold complementary perceptions of complex social-ecological interdependencies. Our approach of measuring between group cognitive distances ensures that we bring in adequate knowledge diversity from across multiple stakeholder groups to harness their collective intelligence. Nevertheless, we did not evaluate whether more diverse groups improve group task performance.

Finally, our findings have applications for designing inclusive processes and adaptive co-management practices [23]. Such approaches encourage the participation and involvement of relevant stakeholders and may enhance the credibility and legitimization of management strategies while resource users, managers, NGOs, policymakers, and scientists bridge their divides and jointly agree on possible management actions for uncertain ecosystems [73]. Furthermore, to achieve knowledge co-production, inclusive processes with buy-in from diverse individuals should also guarantee an increase in the total pool of available knowledge and cognitive coverage. Our study assures that involving diverse groups of stakeholders into adaptive co-management can also achieve knowledge co-production: the “Iterative and collaborative processes involving diverse types of expertise, knowledge and actors to produce context-specific knowledge and pathways towards a sustainable future” [24]. However, it worth noting that inclusion of diverse stakeholder groups with diverging perspectives and knowledge, if not properly harnessed, may undermine the success of co-management and knowledge co-production processes as conflicts may arise. Importantly, dialogue between different stakeholder groups needs to be mediated and stakeholder engagement requires extensive facilitation, such that conflicting representations of the system/problem does not reduce the effectiveness or the value of diversity, but guarantees the creation of between-group synergies.

## Supporting information

S1 File This file includes supporting materials, figures and tables for “Do social identity and cognitive diversity correlate in environmental stakeholders? A novel approach to measuring cognitive distance within and between groups.” (PDF)
