# Supplementary material for: Do social identity and cognitive diversity correlate in environmental stakeholders? A novel approach to measuring cognitive distance within and between groups
Source: PLoS One. 2021 Nov 4;16(11):e0244907. doi: 10.1371/journal.pone.0244907 (PMC8568201; doi:10.1371/journal.pone.0244907)
Supplement: S1 File — (PDF) [file pone.0244907.s001.pdf]

Supporting Information for

Do social identity and cognitive diversity correlate in environmental stakeholders? A novel approach to measuring cognitive distance within and between groups

Payam Aminpour*, Heike Schwermer, and Steven Gray

***** Corresponding Author: pmohamm#3@jhu.edu
Department of Environmental Health and Engineering, Johns Hopkins University, Baltimore, MD 21218, USA.

Journal:
Plos One, Primary research article, Social Science

The case of Western Baltic cod

Cod of the western Baltic Sea (Fig. S1) is not only ecologically of great importance (i.e. predator-prey relationships), also the commercial fishery, which is dependent on this species, shapes the area of the North German Baltic Sea coast and thus provides a high socio-cultural variety. In addition, tourism and especially recreational fisheries are main components of the region, i.e. anglers come from all over Germany to the Baltic Sea coast to catch for cod and thus have a great influence on the characteristics of this region.

However, the state of the stock is currently outside safe biological limits (ICES, 2019). In order to ensure a sustainable management of commercial fish stocks like cod in the Western Baltic Sea, the European Union (EU) has implemented various management measures within the framework of the Common Fisheries Policy (EC, 2013; EC, 2016). These include, in particular, the so-called total allowable catch quotas (i.e. TACs). In the specific case of Western Baltic cod and due to its low stock size, also recreational fisheries are regulated by a fixed daily catch limit since 2017 (i.e. bag-limit).

However, the level of these two management measures is controversially discussed. Stakeholders involved (i.e. managers, scientists, environmental organizations) or directly affected (i.e. commercial fishery, recreational fishery, tourism) have partially strongly divergent perceptions on the cause of the stock status and so of the appropriate measures which need to be taken in terms of stock recovery. This state of affairs continues to these days and has led to hardening fronts between these groups.

In order to investigate how these different perceptions are described and structured, we have collected and analyzed mental models of different stakeholders from 5 groups, i.e. commercial and recreational fisheries, NGOs, tourism, scientific experts and management agencies (Table S1).

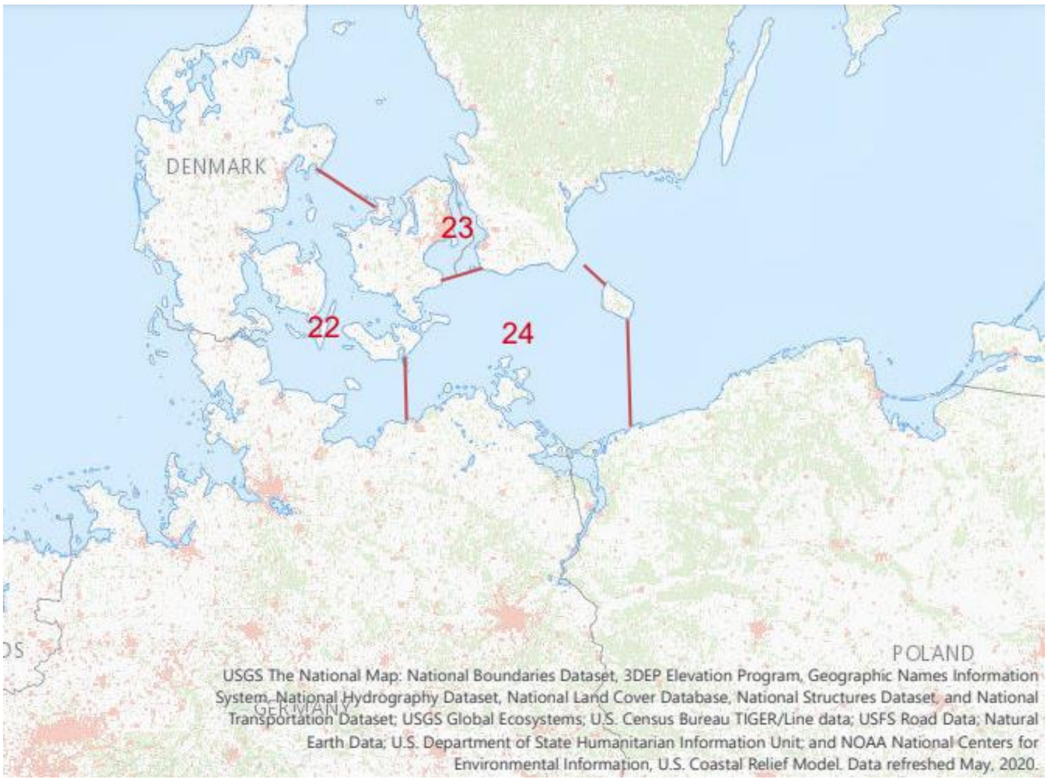

Fig S1. Map of the Baltic Sea region. This map shows the different subdivisions (SD) set by the FAO. The western Baltic Sea and thus the distribution area of Western Baltic cod is represented by SD22-24, i.e. the Belts Sea (SD22), the Sound (SD23), and the Arkona Sea (SD24). Here, the region of interest is the German coast within SD22-24. The map is reprinted from USGS National Map Viewer (Public Domain) under a CC BY license. This map is similar but not identical to the original map by FAO Fisheries Division [online], and is therefore for illustrative purposes only.

Cognitive map elicitation protocol

We elicited stakeholders' mental models using a five-step protocol (see Schwermer et al. 2021 for more detail). First, participants were given a handout to prepare for the interview 1 week in advance (Step 1). To avoid misunderstanding, again the handout was explained in detail before stakeholders' mental models were created (Step 2). These steps were followed by an identification of the system components and their causal relationships by the participants (Step 3), from which they then drew a concept map representing their mental model following routine FCM data collection practices with open-ended concepts (Step 4). These maps were normalized and digitized after the interview and sent back to the interviewees for validation (Step 5).

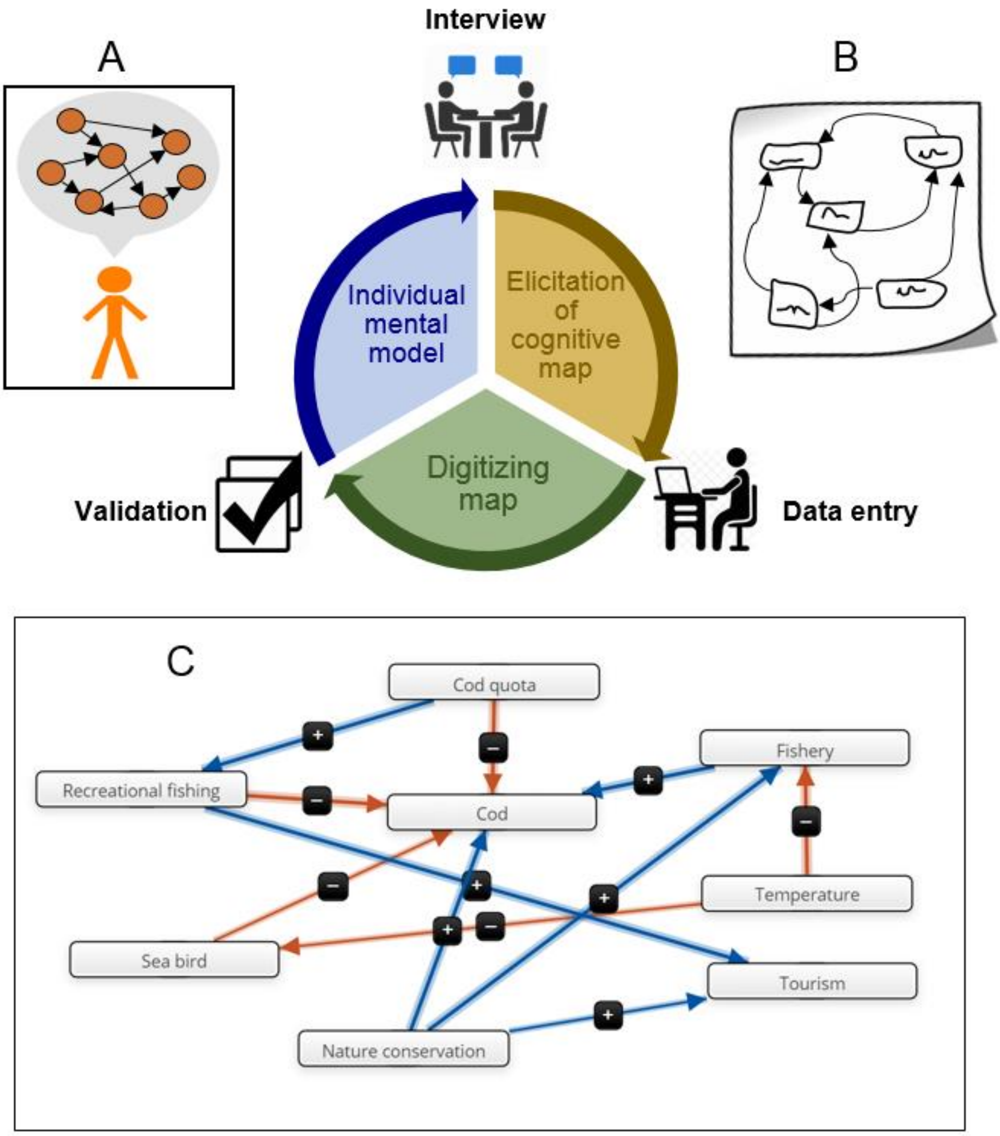

Fig S2. Cognitive map elicitation. (A) shows how an individual constructed a mental model in their head. They then participated in an interview process where individuals were asked to identify relevant concepts (i.e., system components) and their causal relationships, from which they then drew a concept map representing their mental models about Western Baltic cod ecosystem and fisheries management (B). Participants' cognitive maps were digitized after the interview (i.e. maps were converted to digital weighted directed graphs using www.mentalmodeler.org) (C) and sent back to the interviewees for validation.

In order to evaluate the perception of the social-ecological system (SES) of the cod fishery in the western Baltic Sea from the perspective of different stakeholders, participants from 6 relevant groups were interviewed.

Table S1. Description of stakeholder groups. (NGO=non-governmental organization, Com Fish=commercial fishery representatives, MV=Mecklenburg-Western Pomerania, Rec Fish=recreational fishery representatives, SH=Schleswig-Holstein).

Stakeholder group	Description	%
NGO	Representatives of i) marine protection of the Baltic Sea at regional, national and international level, ii) local fisheries in SH and iii) certification of commercial fisheries	18.2
Com Fish	Representatives of commercial fisheries at national and state level, manager of fishing cooperative, commercial fishers from MV and SH	21.2
Managers	Officials focusing on i) catch quotas, ii) fisheries management at international and national level, and iii) nature conservation as well as iv) angling tourism at regional level	18.2
Rec Fish	Representative of the recreational fisheries at i) national and ii) state level and iii) with focus on sea angling	12.1
Scientists	Academics with research focus on i) economy of commercial fisheries, ii) Baltic fish ecology, iii) Baltic fisheries management and iv) gear development in fisheries	18.2
Tourism	Members of tourism associations at regional level with focus on i) the promotion of regional angling tourism or ii) tourism activities in nature, as well as iii) manager of a fishing store	12.1

Additional references

EC (2013). Regulation (EU) No 1380/2013 of the European Parliament and of the council of 11 December 2013 on the Common Fisheries Policy, amending Council Regulations (EC) No 1954/2003 and (EC) No 1224/2009 and repealing Council Regulations (EC) No 2371/2002 and (EC) No 639/2004 and Council Decision 2004/585/EC

EC (2016). Regulation (EU) 2016/1139 of the European Parliament and of the Council of 6 July 2016 establishing a multiannual plan for the stocks of cod, herring and sprat in the Baltic Sea and the fisheries exploiting those stocks, amending Council Regulation (EC) No 2187/2005 and repealing Council Regulation (EC) No 1098/2007.

FAO (2020). FAO Major Fishing Areas. Atlantic, Northeast (Major Fishing Area 27). http://www.fao.org/fishery/area/Area27/en#FAO-fishing-area-27.3.d [29.10.2020]

ICES (2019). ICES Advice on fishing opportunities, catch, and effort Baltic Sea ecoregion. Cod (*Gadus morhua*) in subdivisions 22–24, western Baltic stock (western Baltic Sea). Copenhagen.

Schwermer, H., Aminpour, P., Reza, C., Funk, S., Möllmann, C., & Gray, S. (2021). Modeling and understanding social–ecological knowledge diversity. Conservation Science and Practice, e396.
